# Comparative evaluation of tumor targeting using the anti-HER2 ADAPT scaffold protein labeled at the C-terminus with indium-111 or technetium-99m

**DOI:** 10.1038/s41598-017-15366-w

**Published:** 2017-11-07

**Authors:** Javad Garousi, Sarah Lindbo, Bogdan Mitran, Jos Buijs, Anzhelika Vorobyeva, Anna Orlova, Vladimir Tolmachev, Sophia Hober

**Affiliations:** 1 Institute for Immunology, Genetics and Pathology, Uppsala University, Uppsala, Sweden; 2 School of Biotechnology, Division of Protein Technology, KTH Royal Institute of Technology, Stockholm, Sweden; 3 Division of Molecular Imaging, Department of Medicinal Chemistry, Uppsala University, Uppsala, Sweden

## Abstract

ABD-Derived Affinity Proteins (ADAPTs) is a novel class of engineered scaffold proteins derived from an albumin-binding domain of protein G. The use of ADAPT6 derivatives as targeting moiety have provided excellent preclinical radionuclide imaging of human epidermal growth factor 2 (HER2) tumor xenografts. Previous studies have demonstrated that selection of nuclide and chelator for its conjugation has an appreciable effect on imaging properties of scaffold proteins. In this study we performed a comparative evaluation of the anti-HER2 ADAPT having an aspartate-glutamate-alanine-valine-aspartate-alanine-asparagine-serine (DEAVDANS) N-terminal sequence and labeled at C-terminus with ^99m^Tc using a cysteine-containing peptide based chelator, glycine-serine-serine-cysteine (GSSC), and a similar variant labeled with ^111^In using a maleimido derivative of 1,4,7,10-tetraazacyclododecane-1,4,7,10-tetraacetic acid (DOTA) chelator. Both ^99m^Tc-DEAVDANS-ADAPT6-GSSC and ^111^In-DEAVDANS-ADAPT6-GSSC-DOTA accumulated specifically in HER2-expressing SKOV3 xenografts. The tumor uptake of both variants did not differ significantly and average values were in the range of 19–21%ID/g. However, there was an appreciable variation in uptake of conjugates in normal tissues that resulted in a notable difference in the tumor-to-organ ratios. The ^111^In-DOTA label provided 2–6 fold higher tumor-to-organ ratios than ^99m^Tc-GSSC and is therefore the preferable label for ADAPTs.

## Introduction

Selection of appropriate therapy of disseminated cancer requires information concerning expression of molecular targets in metastases. Currently, such information is obtained routinely by analysis of biopsy samples, which is associated with morbidity and sampling errors. Radionuclide molecular imaging of cancer-associated proteins expressed in malignant cells is attracting an increasing attention as a non-invasive and potentially more accurate alternative to biopsies. Radiolabeled antibodies have successfully been tried in clinics for imaging of cancer cells that overexpress tyrosine kinase receptors^1–3^. Nevertheless, a long residence time of radiolabeled antibodies in blood causes high background and reduces the sensitivity of the imaging^4^. The use of smaller antibody fragments for imaging demonstrated that the contrast can be increased and time between injection and imaging may be shortened^5^. Imaging using single domain antibodies (sdAb), the smallest antibody form, provided good quality images^6,7^. The use of mathematic models predicts that further reduction of imaging probes size should improve imaging contrast even more^8^. However, production of stable immunoglobulin-based imaging probes smaller than sdAb seems to be challenging. An alternative to immunoglobulins might be so called engineered scaffold proteins, small non-immunoglobulin binders selected by using molecular display approaches^9^. The affibody molecule ABY-25 is an example of a scaffold protein that has demonstrated highly sensitive and specific imaging of HER2 expression in disseminated breast cancer^10^.

Recently, a new type of scaffold proteins, ADAPTs, has been developed^11^. ADAPT is derived from the albumin-binding domain (ABD) of streptococcal protein G. The ADAPT scaffold consists of 46 amino acids forming a tree helix bundle. The molecular weight of ADAPT is 5 kDa, i.e. it is approximately three-fold smaller than sdAb and 30-fold smaller than antibodies. Randomization of 11 surface exposed amino acids enabled the creation of a library, which permitted selection of high-affinity binders to tumor necrosis factor-α (TNFα), human epidermal growth factor receptor type 2 (HER2) and human epidermal receptor growth factor type 3 (HER3), all with retained affinity for albumin^12–14^. ADAPT binders are stable, robust and capable of refolding after denaturation. The absence of cysteines in the structure enables thiol-directed site-specific radiolabeling of ADAPTs by incorporation of a single cysteine at a pre-defined position. Binding to albumin increases uptake in blood and slows urinary exertion, which might result in high background during imaging. Therefore, a HER2-binding ADAPT variant (ADAPT6) lacking albumin binding was created^14^. Earlier we have demonstrated the feasibility of specific imaging of epidermal growth factor type 2 (HER2) using ADAPT6 labeled with ^111^In and ^68^Ga in combination with SPECT and PET imaging, respectively^15^.

Monoclonal antibodies are bulky proteins and their biodistribution is determined essentially by their size and the expression of their targets in tumors and normal tissues. In addition, the internalization rate of an antibody-antigen complex influences cellular retention of non-residualizing labels. In the case of small scaffold proteins, even small modification of the surface (by e.g. the use of different chelators for labeling) might have serious effect on targeting properties. Previous experiences with affibody molecules suggest that composition and position of the purification tag^16–19^, chemical properties of the radionuclide^20,21^, the chelator^22^ and the label position in the molecule^23^, all have strong influence on pharmacokinetics of scaffold proteins. Understanding of such influence may enable development of imaging probes with paramount imaging properties. Therefore, we initiated a series of studies concerning the impact of molecular design of ADAPT on the biodistribution. We found that histidine-containing purification tags and the N-terminal sequence have an impact on the hepatic uptake and the blood clearance rate of anti-HER2 ADAPT6^24,25^. In the first studies^15,24,25^, the labels were positioned at the N-terminus and the best contrast was provided by a variant having a histidine-glutamate-histidine-glutamate-histidine-glutamate-aspartate-alanine-asparagine-serine ((HE)_3_DANS) N-terminal sequence^25^. In a later study, we found that site-specific placement of the residualizing ^111^In-DOTA label and the non-residualizing ^125^I-3-iodo-((4-hydroxyphenyl)ethyl)maleimide (^125^I-HPEM) label at the C-terminus of (HE)_3_DANS-ADAPT6 provides radioconjugates with improved biodistribution properties^26^. In the case of ^111^In, C-terminal placement provided significantly (p < 0.05) higher tumor-to-lung, tumor-to-liver, tumor-to-spleen, and tumor-to-muscle ratios at 4 h after injection compared to N-terminal placement. There was no significant difference between majorities of tumor-to-organ ratios for radioiodinated variants at 4 h after injection, but the tumor uptake was ca. two-fold higher for the construct with the C-terminal position of radioiodine.

The generator-produced radionuclide ^99m^Tc remains to be the most frequently used label in nuclear medicine. Its advantages include low price, availability, good spatial resolution of imaging, low absorbed doses and well-studied chemistry^27^. Apparently, this would be an attractive label for a novel imaging agent. We have earlier demonstrated that the use of cysteine-containing peptide-based chelators at the C-terminus enables efficient and stable labeling of anti-HER2 and anti-EGFR affibody molecules^16,28^. An appealing feature of such chelators is that they are inserted in the amino acid sequence of the targeting protein during recombinant production and thereby additional conjugation steps are unnecessary. In addition, it is possible to modify the residualizing properties of the ^99m^Tc label by varying amino acid composition of the peptide chelator^29,30^. It would be reasonable to place such chelator at C-terminus of (HE)_3_DANS-ADAPT6. However, previous studies have demonstrated that the (HE)_3_-tag competes with cysteine-containing chelators for ^99m^Tc = O, and site-specificity of the labeling is lost in such constructs^31^. Therefore, we omitted the (HE)_3_ tag in this investigation and DEAVDANS-ADAPT6 variant was selected for evaluation.

**Figure 1:**
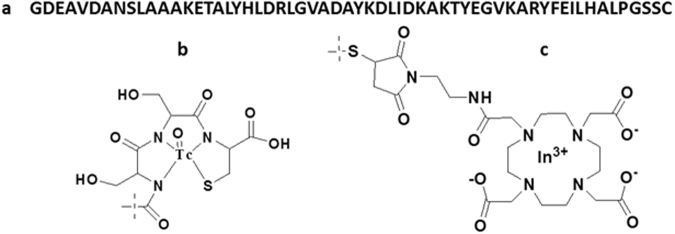
Amino acid sequence of DEAVDANS-ADAPT6-GSSC (**a**) and structure of the labels ^99m^Tc-GSSC (**b**) ^111^In-DOTA-MMA-Cys ^59^ (**c**).

The aim of this study was to evaluate tumor-targeting properties of ^99m^Tc-labeled DEAVDANS-ADAPT6 containing a GSSC chelating sequence at the C-terminus (DEAVDANS-ADAPT6-GSSC) and to compare these properties with properties of a counterpart labeled with ^111^In using maleimido derivative of DOTA conjugated at a C-terminal cysteine (Fig. 1).

## Results

### Production, Purification, Conjugation and Characterization of ADAPT Molecules

**Figure 2:**
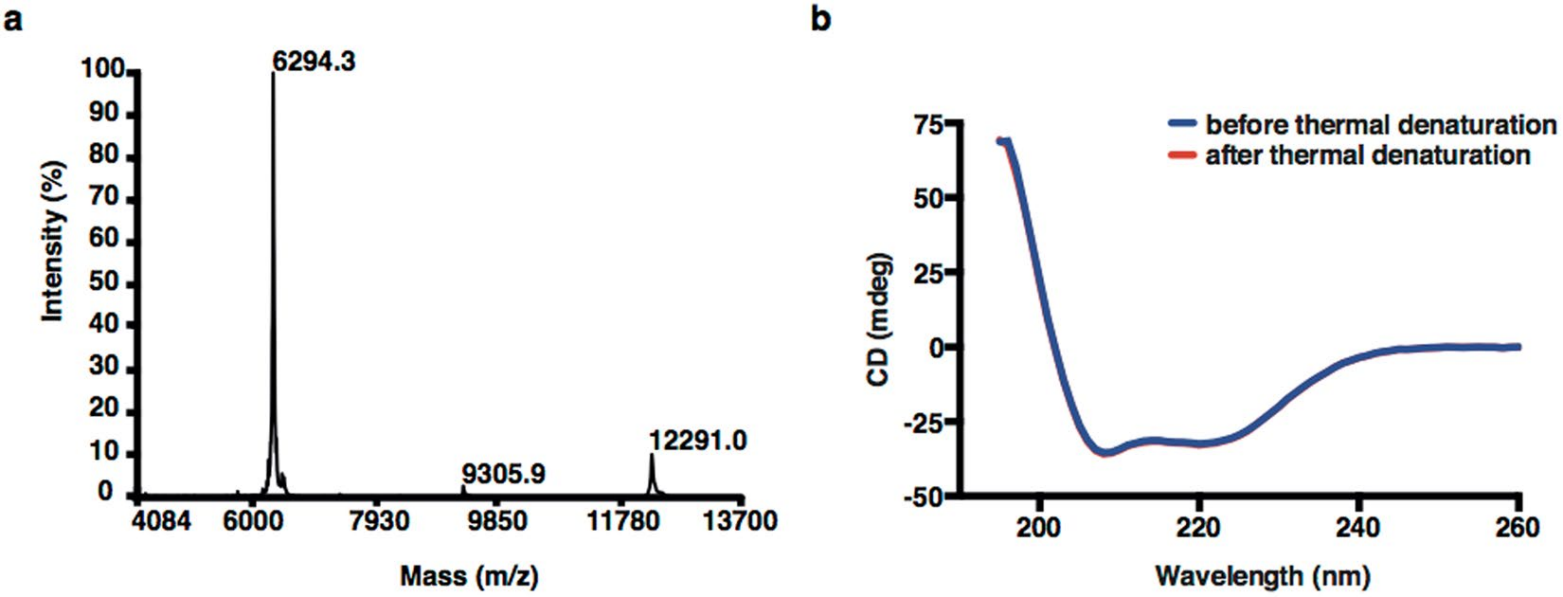
Mass spectrum and secondary structure measurements of DEAVDANS-ADAPT6-GSSC. The molecular weight was measured to 6294.3 Da (**a**) and the construct demonstrated complete refolding after thermal denaturation (**b**).

The construct DEAVDANS-ADAPT6-GSSC was successfully produced in *E*. *coli* and purified using 90 °C heat-treatment followed by ion exchange chromatography. The purity of the construct was above 98%, determined by reversed-phase high-performance liquid chromatography (RP-HPLC), and the identity was confirmed by mass spectrometry. The measured molecular weight was 6294.3 Da, which was in a good agreement with the calculated value of 6292.1 Da (Fig. 2). The protein fraction intended for ^111^In-labeling was successfully conjugated with maleimide-DOTA and the circular dichroism analysis showed high α-helical content, melting temperatures above 65 °C and good refolding properties after heating to 90 °C for both unconjugated and DOTA-conjugated constructs **(**Fig. 2 **)**. The surface plasmon resonance (SPR) measurements demonstrated an affinity to HER2 of 4.0 nM, which is comparable with the parental molecule ADAPT6 (2.5 nM)^15^.

### Labeling

DEAVDANS-ADAPT6-GSSC-DOTA was labeled with ^111^In with the radiochemical yield of 94 ± 5%. The radiochemical purity after purification using NAP-5 size-exclusion column was 99.0 ± 0.5%. Incubation with 500-fold EDTA for 2 h did not show any radionuclide release from the conjugate.

DEAVDANS-ADAPT6-GSSC was labeled with ^99m^Tc with the isolated yield of 31 ± 17%. The radiochemical purity after size-exclusion purification was 98 ± 2%. Incubation with 300-fold cysteine for 4 h showed less than 2% radionuclide release from the conjugate.

### *In Vitro* Binding Specificity and Affinity

**Figure 3:**
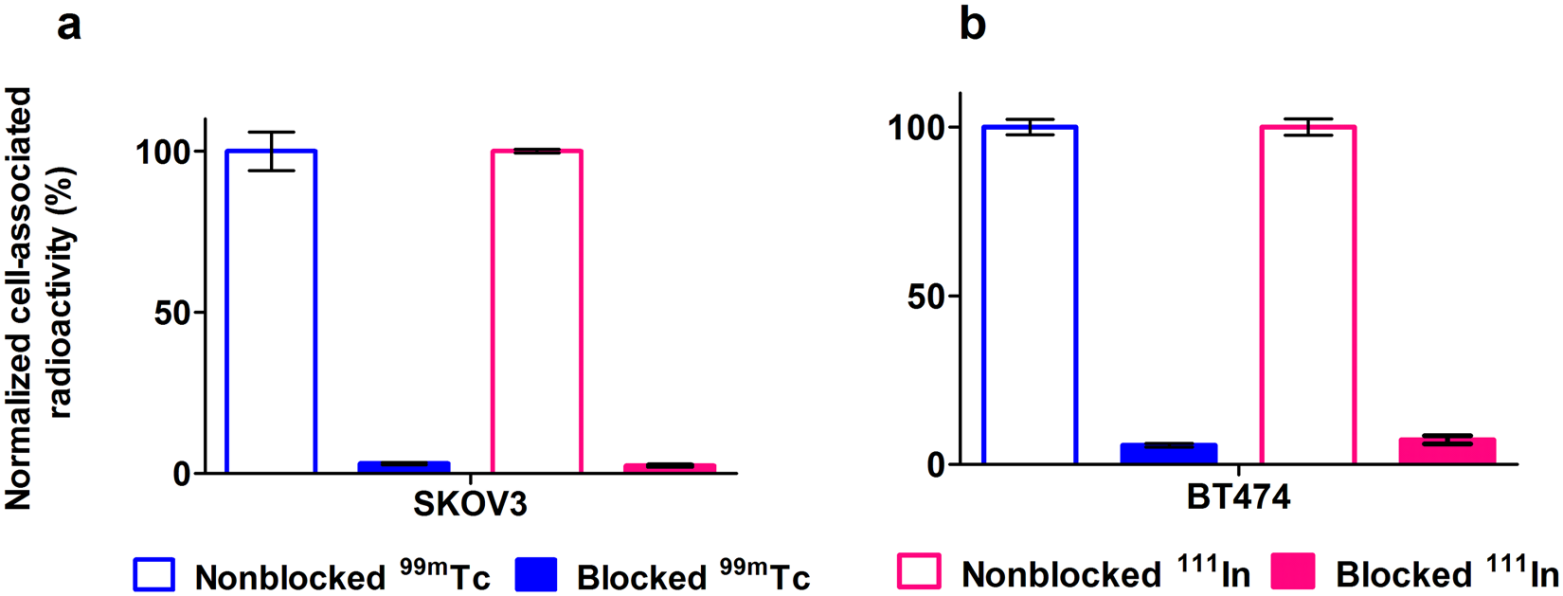
Binding specificity of ^111^In- DEAVDANS-ADAPT6-GSSC-DOTA and ^99m^Tc - DEAVDANS-ADAPT6-GSSC to HER2 expressing SKOV3 cells (**a**) and BT474 cells (**b**). A 100-fold molar excess of non-labeled corresponding variant was used for blocking of the receptors. Data are presented as average of triplicates with SD.

HER2-binding specificity was tested for both radioconjugates using HER2-expressing SKOV3 and BT474 cells. Significant reduction (p < 0.0005) of the binding of both ^111^In- DEAVDANS-ADAPT6-GSSC-DOTA and ^99m^Tc-DEAVDANS-ADAPT6-GSSC to HER2-expressing cells in pre-saturated group compared to non-saturated one confirmed HER2 specific binding (Fig. 3).

InteractionMap evaluation of the LigandTracer measurements suggested two types of interactions of the radiolabeled ADAPTs with HER2. The association rates are similar in both interactions while the dissociation rates are different. Table 1 shows the equilibrium dissociation constant (K_D_) values for the possible interactions between ligands and the HER2 target.

**Table 1:** K_D_ values for the interaction of radioconjugated ADAPT6 with HER2-expressing SKOV3 cells.

	^111^In-DEAVDANS-ADAPT6-GSSC-DOTA	^99m^Tc-DEAVDANS-ADAPT6-GSSC
K_D1_	32.3 ± 0.5 pM	174 ± 48 pM
K_D2_	1.31 ± 0.1 nM	1.6 ± 0.3 nM

The values have been calculated using InteractionMap software.

**Figure 4:**
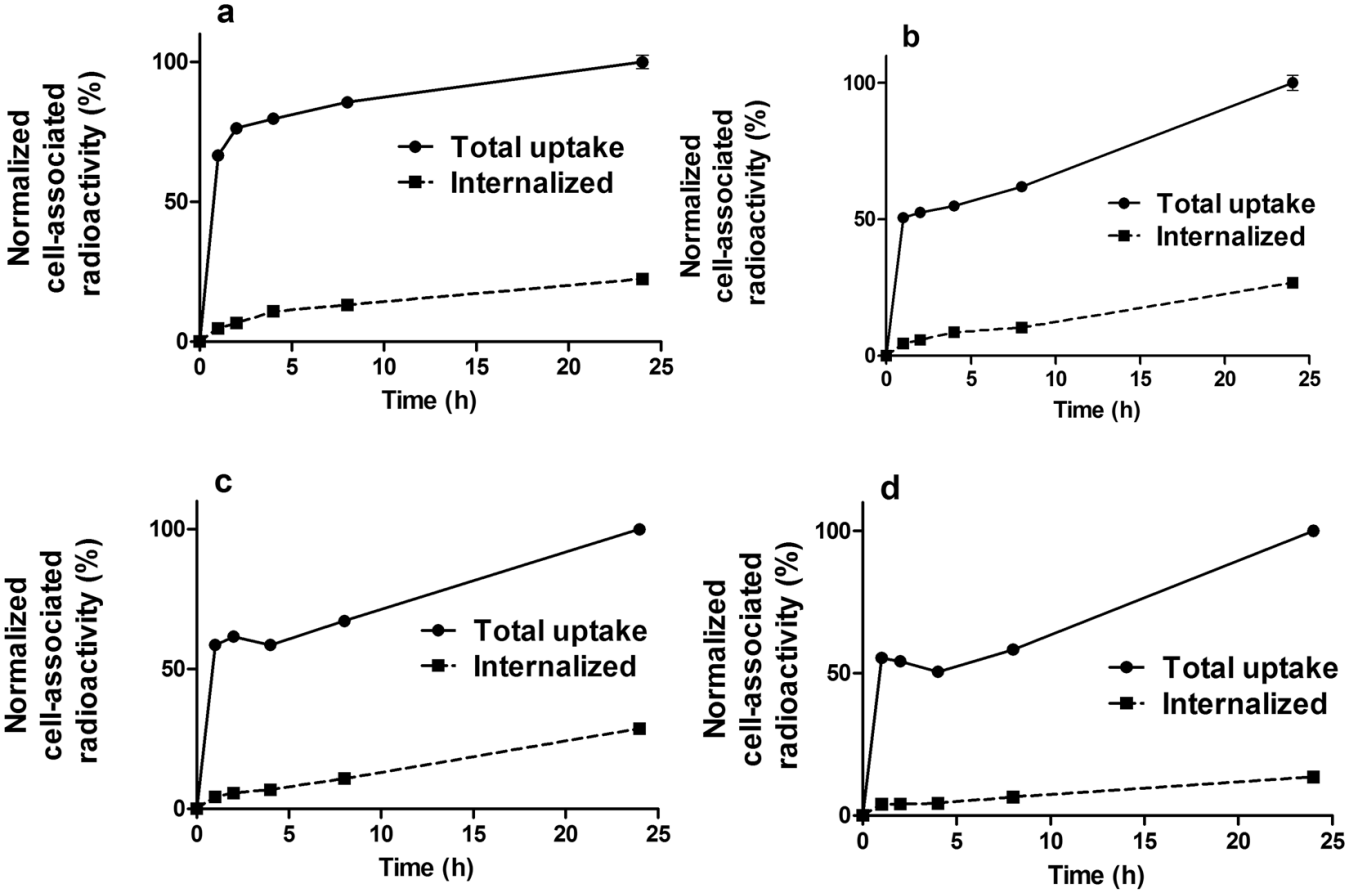
Cellular processing of ^99m^Tc-DEAVDANS-ADAPT6-GSSC (**a** and **b**) ^111^In- DEAVDANS-ADAPT6-GSSC-DOTA (**c** and **d**) after binding to HER2 expressing SKOV3 (**a** and **c**) and BT474 (**b** and **d**) cells. Data are presented as average of triplicates with SD. Error bars are not visible for all time points because they are smaller than the symbols.

The processing of ^111^In-DEAVDANS-ADAPT6-GSSC-DOTA and ^99m^Tc-DEAVDANS-ADAPT6-GSSC bound to HER2 expressing SKOV3 and BT474 cells is presented in Fig. 4. The pattern of the processing was quite similar for both conjugates. For both cell lines binding was shown to be rapid and internalization slow. The internalized fractions after 24 h of incubation for both cell lines were lower than 30% of the total cell bound radioactivity.

### *In Vivo* Studies

Tumor targeting, *in vivo* HER2 binding specificity and biodistribution of both radioconjugates was evaluated in BALB/C nu/nu mice bearing SKOV3 xenografts.

**Figure 5:**
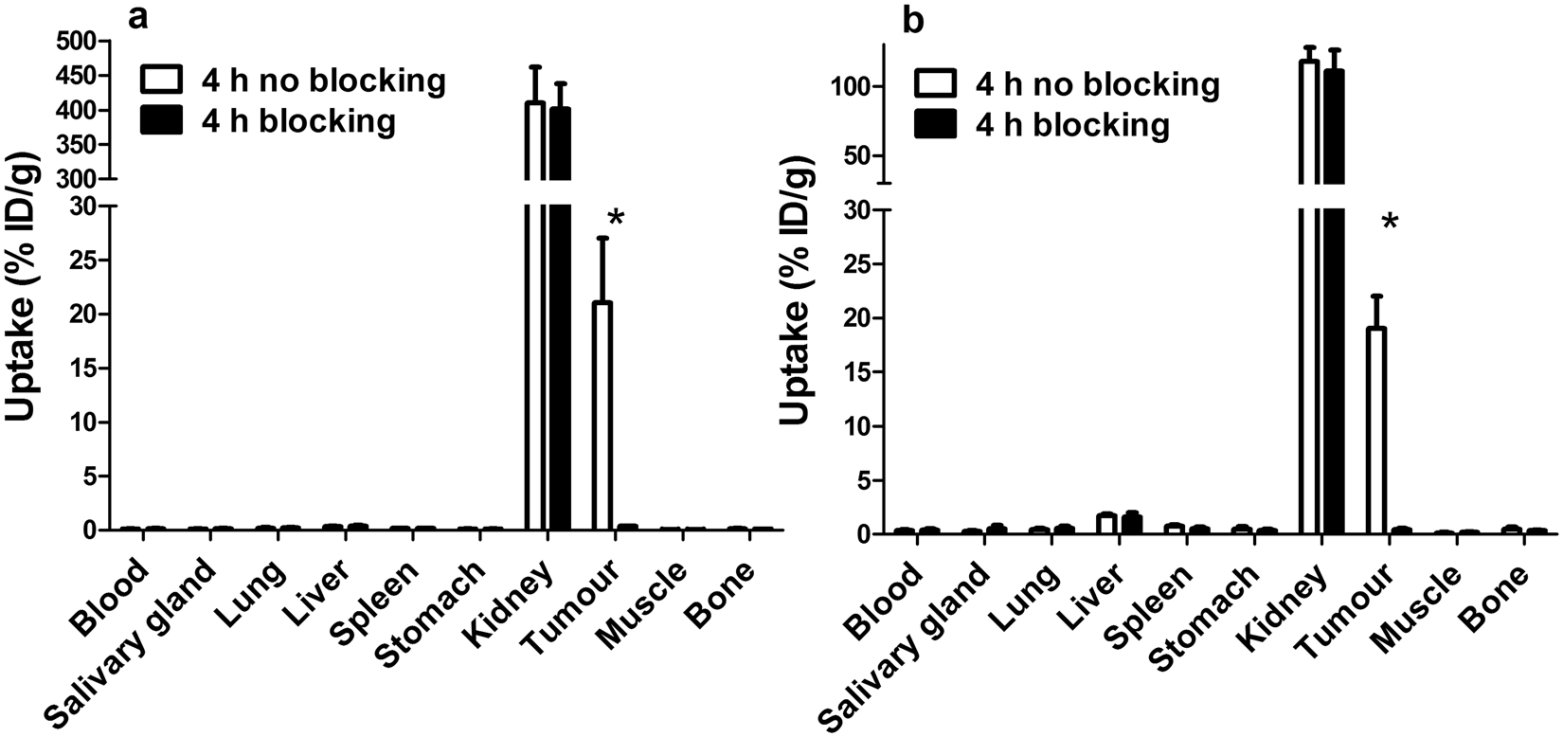
*In vivo* specificity of ^111^In-DEAVDANS-ADAPT6-GSSC-DOTA (**a**) and ^99m^Tc-DEAVDANS-ADAPT6-GSSC (**b**) to tumors in BALB/C nu/nu mice bearing SKOV3 xenografts at 4 hours p.i. 24 h before injection of tracers 15 mg of trastuzumab was injected per mouse in control groups to saturate HER2 receptors. The data are presented as the average (%ID/g) from four animals ± SD. Asterisk marks a significant difference between uptake with and without trastuzumab treatment.

The results of *in vivo* specificity tests confirmed HER2-specific uptake of ^111^In- DEAVDANS-ADAPT6-GSSC-DOTA and ^99m^Tc-DEAVDANS-ADAPT6-GSSC in SKOV3 tumors. Pre-saturation of HER2 in SKOV3 xenografts with anti-HER2 antibody trastuzumab significantly decreased (p < 0.00005) the tumor-associated radioactivity from 21 ± 6 to 0.33 ± 0.04%ID/g for ^111^In-DEAVDANS-ADAPT6-GSSC-DOTA and from19 ± 3 to 0.4 ± 0.1 for ^99m^Tc-DEAVDANS-ADAPT6-GSSC (Fig. 5).

The results of biodistribution measurements of ^111^In-DEAVDANS-ADAPT6-GSSC-DOTA and ^99m^Tc-DEAVDANS-ADAPT6-GSSC at 1 and 4 h after injection in mice bearing SKOV3 xenografts are presented in Tables 2 and 3. The data reflect the common features of ADAPTs, such as rapid decrease of blood-born radioactivity, low radioactivity accumulation in intestines and their content, and high renal re-absorption. The average tumor uptake was on the level of 18–21%ID/g for both conjugates. There was no significant difference between tumor uptakes of ^99m^Tc- and ^111^In-labeled ADAPT6 at any time point. The tumor uptake of each conjugate did not differed significantly at 1 h and 4 h after injection. However, the uptake of ^111^In-DEAVDANS-ADAPT6-GSSC-DOTA and ^99m^Tc-DEAVDANS-ADAPT6-GSSC in normal tissues demonstrated appreciable difference. In kidneys, uptake of ^111^In-DEAVDANS-ADAPT6-GSSC-DOTA was about 410%ID/g at both time points, while for ^99m^Tc-DEAVDANS-ADAPT6-GSSC it was 312% ID/g after one hour and decreased to 118%ID/g at 4 p.i. In the case of ^99m^Tc-DEAVDANS-ADAPT6-GSSC, the renal uptake was significantly (p < 0.05) lower than uptake of ^111^In-labeled variant already at 1 h after injection and decreased nearly three-fold at 4 h after injection. The uptake of ^111^In labeled ADAPT6 in all other normal tissues was significantly lower than the uptake of ^99m^Tc - labeled one.

**Table 2:** Biodistribution of ^111^In-DEAVDANS-ADAPT6-GSSC-DOTA and ^99m^Tc-DEAVDANS-ADAPT6-GSSC molecules in BALB/C nu/nu mice bearing SKOV3 xenografts at 1 and 4 h after injection.

	1 h		4 h	
	^111^In-DEAVDANS-ADAPT6-GSSC-DOTA	^99m^Tc-DEAVDANS-ADAPT6-GSSC	^111^In-DEAVDANS-ADAPT6-GSSC-DOTA	^99m^Tc-DEAVDANS-ADAPT6-GSSC
blood	0.4 ± 0.1^a^	1.2 ± 0.1^a,b^	0.10 ± 0.02^b^	0.34 ± 0.08^b^
salivary gland	0.18 ± 0.06	0.6 ± 0.1^a,b^	0.10 ± 0.02^b^	0.27 ± 0.05^b^
lung	0.5 ± 0.1^a^	1.2 ± 0.1^a,b^	0.19 ± 0.04^b^	0.41 ± 0.15^b^
liver	0.26 ± 0.07	1.9 ± 0.2^b^	0.31 ± 0.05^b^	1.7 ± 0.2^b^
spleen	0.21 ± 0.05	0.82 ± 0.04^b^	0.14 ± 0.03^b^	0.8 ± 0.1^b^
stomach	0.2 ± 0.1	1.0 ± 0.5^b^	0.10 ± 0.02^b^	0.5 ± 0.3^b^
kidney	415 ± 67	312 ± 12^a,b^	410 ± 52^b^	118 ± 10^a,b^
tumour	18 ± 4	19 ± 3	21 ± 6	19 ± 3
muscle	0.10 ± 0.03^a^	0.33 ± 0.09^a,b^	0.05 ± 0.01^b^	0.13 ± 0.03^b^
bone	0.20 ± 0.07	0.75 ± 0.07^a,b^	0.11 ± 0.04^b^	0.47 ± 0.17^ab^
GI tract	2.3 ± 0.7	1.6 ± 0.4	1.5 ± 0.5	1.9 ± 0.99
carcass	0.6 ± 0.1	10 ± 4^b^	0.09 ± 0.03 ^b^	6 ± 2^b^

Results are presented as an average %ID/g and SD of 4 animals.Data for gastrointestinal tract with content and carcass are presented as %ID/whole sample.

^a^significant difference between uptake of tracer at 1 and 4 h after injection;

^b^significant difference between uptake of conjugate labeled with ^111^In and ^99m^Tc at the same time point**;**

**Table 3:** Tumor-to-organ ratio of ^111^In-DEAVDANS-ADAPT6-GSSC-DOTA and ^99m^Tc-DEAVDANS-ADAPT6-GSSC in BALB/C nu/nu mice bearing SKOV3 xenografts at 1 and 4 h after injection.

	1 h		4 h	
	^111^In-DEAVDANS-ADAPT6-GSSC-DOTA	^99m^Tc-DEAVDANS-ADAPT6-GSSC	^111^In-DEAVDANS-ADAPT6-GSSC-DOTA	^99m^Tc-DEAVDANS-ADAPT6-GSSC
blood	49 ± 6^a,b^	15 ± 2^a,b^	206 ± 15^a,b^	54 ± 4^a,b^
salivary gland	104 ± 21^a,b^	28 ± 7^a,b^	225 ± 54^a,b^	64 ± 8^a,b^
lung	38 ± 5^a,b^	15 ± 2^a,b^	114 ± 20^a,b^	47 ± 7^a,b^
liver	69 ± 4^b^	10 ± 1^b^	68 ± 11^b^	11 ± 1^b^
spleen	87 ± 26^a,b^	23 ± 4^b^	148 ± 17^a,b^	25 ± 2^b^
stomach	100 ± 29^a,b^	22 ± 9^a,b^	210 ± 34^a,b^	46 ± 16^a,b^
kidney	0.04 ± 0.01^b^	0.06 ± 0.01^a,b^	0.05 ± 0.01^b^	0.16 ± 0.03^a,b^
muscle	181 ± 20^a,b^	57 ± 12^b^	472 ± 93^a,b^	134 ± 21^b^
bone	99 ± 30^a,b^	25 ± 2^a,b^	210 ± 31^a,b^	42 ± 10^a,b^

Results are presented as an average %ID/g and SD of 4 animals.Data for gastrointestinal tract with content are presented as %ID/whole sample.

^a^significant difference between uptake of tracer at 1 and 4 h after injection;

^b^significant difference between uptake of conjugate labeled with ^111^In and ^99m^Tc at the same time point;

Tumor-to-organ ratios of ^111^In-DEAVDANS-ADAPT6-GSSC-DOTA were significantly (p < 0.05) higher at both 1 and 4 h after injection for all organs except kidneys (Table 3). At 4 h after injection, the use of ^111^In-label provided approximately six-fold higher tumor-to-organ ratios for liver and spleen, approximately five-fold higher for stomach, and four-fold for blood and salivary gland. The tumor-to-lung ratio was approximately two-fold higher for ^111^In at this time point.

Analysis of the blood-born radioactivity 1 h after injection of ^99m^Tc-DEAVDANS-ADAPT6-GSSC showed that 37 ± 6% of the total radioactivity was presented in the form of radiometabolites with the molecular weight of less than 5 kDa.

**Figure 6:**
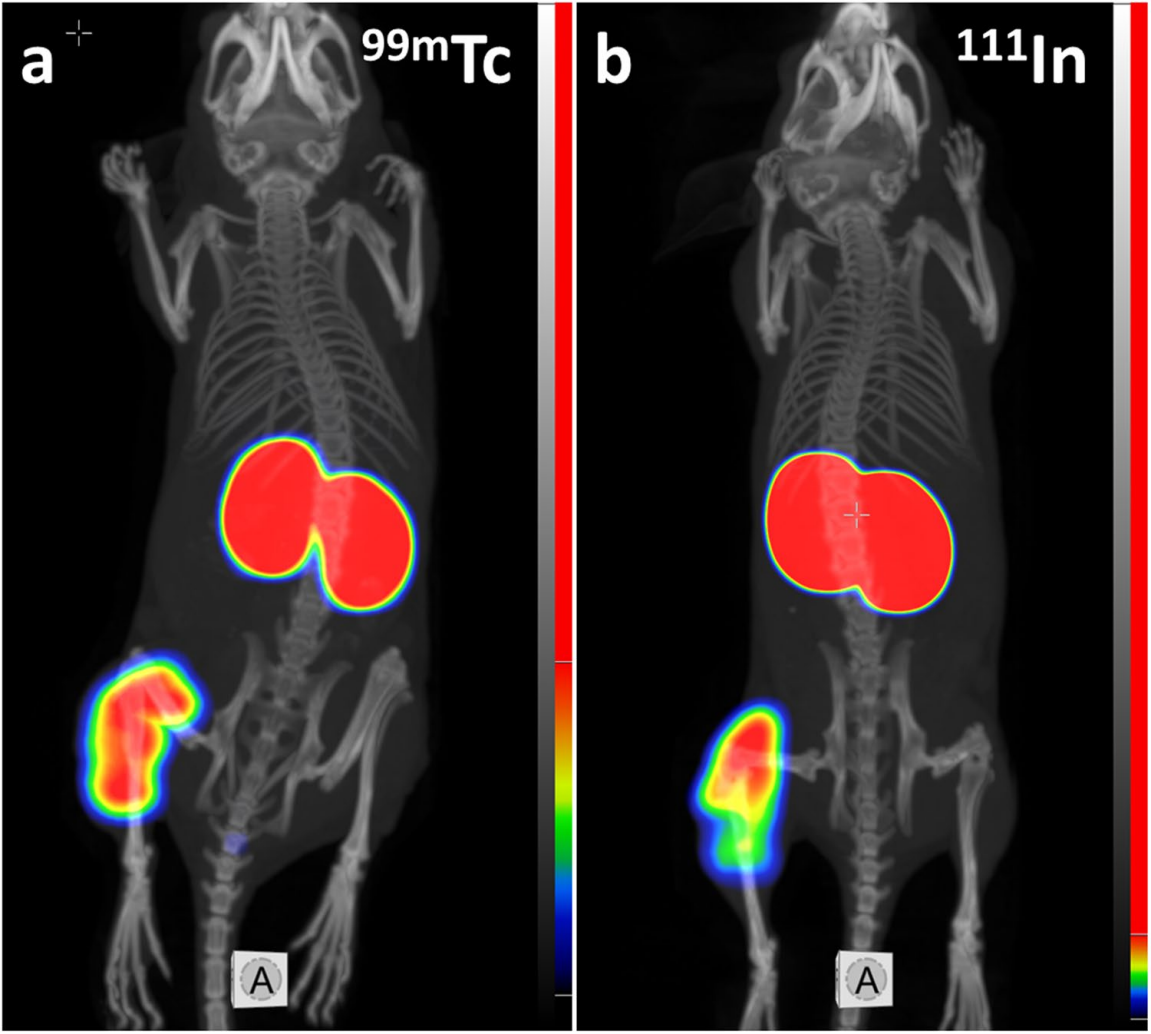
MicroSPECT/CT imaging of HER2-expressing SKOV3 xenografts (left hind legs) at 4 h after injection of ^99m^Tc-DEAVDANS-ADAPT6-GSSC (**a**) and ^111^In-DEAVDANS-ADAPT6-GSSC-DOTA (**b**). Color scales were adjusted to provide clear visualization of tumors.

Experimental imaging confirmed that both ^111^In-DEAVDANS-ADAPT6-GSSC-DOTA and ^99m^Tc-DEAVDANS-ADAPT6-GSSC are able to visualize HER2-expressing xenografts in mice (Fig. 6). The images were in agreement with the *ex vivo* measurements and demonstrated high uptake in kidneys and low uptake in other tissues.

## Discussion

The combination of radionuclide, chelator and required labeling chemistry are factors that can have very strong impact on the contrast provided by a protein-based imaging probe. Due to the complexity of *in vivo* interactions it is still impossible to predict an optimal labeling approach, and therefore experimental optimization is required to collect sufficient information for a rational design of imaging agents. Here, cysteine-containing peptide based chelators have been selected for evaluation because this class of chelators provided stable labeling of affibody molecules with technetium-99m^29,30,32^ and rhenium-188^33^ when placed at C-terminus. However, this study showed that the site-specificity of labeling probably was not perfect in the case of DEAVDANS-ADAPT6-GSSC since a fraction of ^99m^Tc could be released by challenging with a large excess of cysteine. Most likely, some amino acids with electron-donating side chains form a weak chelating pocket and thereby compete with the GSSC sequence. *In vivo*, this fraction might be transchelated to blood proteins, which can slow the clearance of radioactivity from blood. However, this weakly bound technetium could be stripped by free cysteine and a pre-purification cysteine challenge seems to solve this problem providing a stable conjugate. Unfortunately, this is associated with a loss of isolated radiochemical yield of ^99m^Tc-DEAVDANS-ADAPT6-GSSC. Importantly, ^99m^Tc-DEAVDANS-ADAPT6-GSSC retained the capacity of specific binding to HER2-expressing cell lines (Fig. 3) with high affinity (Table 1). Kinetics of binding to living cells showed, as before, two types of binding with equally high one-rate but different off-rates. This effect has been observed for affibody molecules and ADAPTs binding receptor tyrosine kinases and is most likely associate with conformation changes due to homo- and heterodimerization of the receptors^24–26,32^.

Targeting specificity has been confirmed *in vivo* (Fig. 5). ADAPT6 binds to the same epitope as the anti-HER2 antibody trastuzumab^15^. Significant reduction of both ^111^In-DEAVDANS-ADAPT6-GSSC-DOTA and ^99m^Tc-DEAVDANS-ADAPT6-GSSC accumulation in HER2-expressing xenografts after pre-saturation with trastuzumab demonstrated targeting specificity beyond any reasonable doubts. Of note, accumulation in saturated xenografts was on the level of 1.5–2% compared with non-saturated ones. This showed that unspecific accumulation of ADAPT6 due to enhanced permeability and retention (EPR) effect was very low and the risk of false positive findings was negligible.

The biodistribution studies revealed a dramatic difference between renal retention of ^111^In-DEAVDANS-ADAPT6-GSSC-DOTA and ^99m^Tc-DEAVDANS-ADAPT6-GSSC (Table 2). This effect might be explained by difference in residualizing properties of the ^111^In-DOTA and the ^99m^Tc-GSSC labels. The renal reabsorption of peptides is associated with a rapid internalization, translocation to lysosomal compartment and degradation^34^. Radiometabolites of residualizing radiometal labels (such as ^111^In-DOTA) get trapped in the lysosomes, which cause long renal retention of radionuclide. Radiometabolites of non-residualizing labels diffuse through lysosomal and cellular membranes and leak from proximal tubuli cells. This is associated with a rapid decrease of renal radioactivity. Apparently, residualizing properties of ^99m^Tc-GSSC are much weaker than those of ^111^In-DOTA. This is in agreement with our previous findings with affibody molecules, where the use of homologous ^99m^Tc-GGSC^29^, ^99m^Tc-GSGC^30^ and ^99m^Tc-maSSS^35^ labels was associated with much lower renal retention compared to the ^111^In-DOTA label^22^.

The tumor uptake of ^99m^Tc-DEAVDANS-ADAPT6-GSSC was at the same level at 1 and 4 h after injection (Table 2) and did not differ significantly from tumor uptake of ^111^In-DEAVDANS-ADAPT6-GSSC-DOTA. This means that the residualizing properties of the labels were not essential for retention of radioactivity in tumors. This is in an agreement with the results of cellular processing experiment (Fig. 4), which demonstrated slow internalization of DEAVDANS-ADAPT6-GSSC after binding to HER2-expressing malignant cells. In this case, cellular retention of a radionuclide depends more on the off-rate of the targeting probe than on residualizing properties of the label.

The major effect of the non-residualizing properties of ^99m^Tc-GSSC was observed in the concentration of radioactivity in the blood and in the normal organ uptake. At both time points, the uptake of ^99m^Tc was appreciably higher in all normal organs except for kidneys than the uptake of ^111^In. Analysis of blood-born radioactivity showed that ca. 40% of radioactivity was associated with low-molecular-weight radiometabolites, which is a much higher proportion than <10%, which are typical for ^111^In-DOTA-labeled ADAPTs^26^. This effect was most likely not because of release of ^99m^Tc-pertechnetate (TcO_4_^−^), as the uptake in salivary gland and stomach was not very high. More probably, renal radiometabolites returned to blood and were redistributed to other tissues. This resulted in substantially lower tumor-to-organ ratios for ^99m^Tc-DEAVDANS-ADAPT6-GSSC compared to ratios of ^111^In-DEAVDANS-ADAPT6-GSSC-DOTA (Table 3). It has to be noted that even with the suboptimal ^99m^Tc-GSSC label, ^99m^Tc-DEAVDANS-ADAPT6-GSSC provided much higher tumor-to-organ ratios compared with monoclonal antibodies. For example, tumor-to-blood, tumor-to-liver and tumor-to-lung ratios in murine models have been shown to be below 10 even at 72 h for any HER2-targeting antibody^36^.

**Figure 7:**
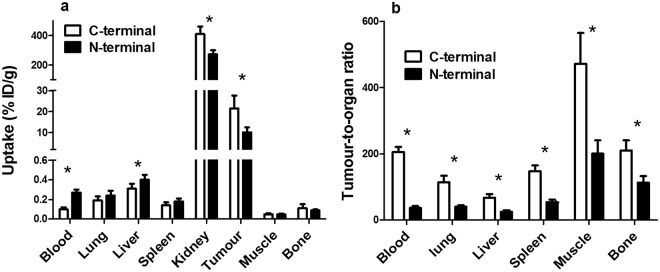
Comparison of ^111^In-DEAVDANS-ADAPT6-GSSC-DOTA (C-terminal label) and ^111^In- DOTA-DEAVDANS-ADAPT6 (N-terminal label). (**a**) Biodistribution and (**b**) tumor-to-organ ratios at 4 h after injection in BALB/C nu/nu mice bearing SKOV3 xenografts. Data for ^111^In-DOTA-DEAVDANS-ADAPT6 are taken from^25^.

Recently, we have found that C-terminal placement of the ^111^In-DOTA label at ADAPT6 with the (HE)_3_DANS N-terminal sequence^26^ provides appreciable improvement of tumor-to-lung, tumor-to-liver, tumor-to-spleen, and tumor-to-muscle ratios compared to N-terminal placement. Such comparison would be of interest for DEAVDANS-ADAPT6 as well. Earlier, we have evaluated biodistribution of ^111^In-DOTA-DEAVDANS-ADAPT6, where DOTA was conjugated to an N-terminal cysteine^25^. Mice used in that study were from the same breeder, SKOV3 cells for xenografts were from the same supplier, and the study was performed by the same personnel using the same protocol. Therefore, comparison should be quite reliable although the experiments were not performed using the same batch of tumor-bearing mice. Comparison (Fig. 7) demonstrated that ^111^In-DEAVDANS-ADAPT6-GSSC-DOTA had appreciably lower blood radioactivity concentration and hepatic uptake, but higher tumor uptake (21 ± 6 vs 10.1 ± 2.4%ID/g) compared to ^111^In-DOTA-DEAVDANS-ADAPT6. We have earlier found that a decrease of local hydrophobicity of affibody molecules resulted in lower hepatic uptake and lower radioactivity levels in the blood^21^. The ^111^In-DOTA complex is hydrophilic and its conjugation decreases local hydrophobicity. In the case of an N-terminal placement, ^111^In-DOTA is placed next to hydrophilic DEAVDANS sequence, and therefore its contribution is insignificant. At the same time, the lipophilicity of the C-terminal ILHALP sequence is not compensated. In DEAVDANS-ADAPT6-GSSC-DOTA two additional hydrophilic serines placed close to the lipophilic C-terminus and the additional conjugation of DOTA enhanced the hydrophilicity of the construct. This might reduce the unwanted binding of ^111^In-DEAVDANS-ADAPT6-GSSC-DOTA to blood proteins, resulting in better extravasation into tumors and faster clearance of the unbound tracer. It has to be noted that chemical features of radiometabolites also should be taken into account. Degradation of polypeptide probes by proteinases might result in metabolites containing a radiolabeled chelate with several adjacent amino acid residues. Since the N-terminal and C-terminal amino acid sequences of the ADAPTs probe are different, the characteristics of the released radiometabolites would also be different. This may be another explanation for the difference in accumulation of radioactivity, especially in normal organs.

In conclusion, placement of an ^111^In-DOTA label at the C-terminus of DEAVDANS-ADAPT6 provides appreciably higher tumor-to-organ ratios than the same placement of a ^99m^Tc-GSSC label. This should provide better sensitivity in imaging of small metastases. Therefore, a C-terminal ^111^In-DOTA label would be the preferential label for ADAPT6.

## Methods

^111^InCl_3_ (in 0.05 M HCl) was purchased from Mallinckrodt Sweden AB (Stockholm, Sweden).^99m^TcO_4_^−^ was obtained by elution of ^99^Mo/^99m^Tc generator (Mallinckrodt, Petten, the Netherlands) with sterile saline solution. All conjugation and labeling buffers were prepared in MQ-water and metal impurities were removed using Chelex^®^ 100 resin (Bio-Rad Laboratories, Hercules, CA, USA). Two different HER2-expressing cell lines, SKOV3 and BT474, used in this study were purchased from American Type Culture Collection (ATCC). RPMI medium containing 10% fetal calf serum, PEST (penicillin 100 IU/ml and 100 μg/ml streptomycin) and 1% L-glutamine was used for cell culture. Radioactivity was measured by an automated γ-spectrometer with a NaI(Tl) detector (1480 Wizard; Wallac Oy, Turku, Finland). GraphPad Prism (version 4.00 for Windows; GraphPad Software) was used to analyze cellular uptake and biodistribution data using unpaired 2-tailed t-test.

### Production, Purification, Conjugation and Characterization of ADAPT Molecules

The gene encoding ADAPT6 was PCR amplified using primers introducing the terminal extension sequences, GDEAVDANS in the N-terminal and GSSC in the C-terminal. The new construct was denoted DEAVDANS-ADAPT6-GSSC. Production, purification, conjugation and characterization were performed according to methods described earlier^25^. Briefly, the DNA fragment was subcloned into an expression vector containing an inducible T7 promoter. Soluble protein was produced in *Escherichia coli* and purified using 90 °C heat-treatment followed by 1 h incubation with 5 mM dithiothreitol (DTT) at 37 °C to reduce the thiol group and avoid dimerization of the protein. The reduced sample was loaded in an anion exchange column (Resource Q, GE Healthcare, Uppsala, Sweden) and eluted using a 0–0.5 M NaCl gradient. The purity of the protein was analyzed by sodium dodecyl sulfate polyacrylamide gel electrophoresis (SDS-PAGE) and RP-HPLC using an analytical column (Zorbax, 300 SB-C18 4.6 × 150 mm, 3.5 µm particle size). The molecular weight of the construct was measured by matrix assisted laser desorption ionization (MALDI) using a 4800 MALDI TOF/TOF analyzer (SCIEX). The construct intended for ^111^In labeling was conjugated with maleimide-DOTA (Macrocyclics) to the thiol group of the C-terminal cysteine and designated DEAVDANS-ADAPT6-GSSC-DOTA. Excess DOTA and unconjugated protein were removed using a semipreparative RP-HPLC column (Zorbax, 300SB-C1, 9.4 × 250 mm, 5 μm particle size, Agilent). The secondary structure, melting temperature and refolding properties were determined by circular dichroism (CD) using a JASCO J-810 spectropolarimeter (JASCO) and the binding strength to HER2 was measured and analyzed using a Biacore T200 instrument and the Biacore T200 evaluation software 2.0 (GE Healthcare).

### Labeling

#### *Labeling of DEAVDANS-ADAPT6-GSSC with*^*99m*^*Tc*

Labeling was performed using a freeze-dried kit, containing 5 mg sodium α-D-glucoheptonate (Celsus Laboratories, Geel, Belgium), 75 µg SnCl_2_ (Fluka Chemika, Buchs, Switzerland) and 100 μg of EDTA (Sigma-Aldrich, Munich, Germany), as described earlier^37^. The lyophilized ADAPT6 (50 µg) was reconstituted in 50 µL degassed PBS and the solution was added to the content of one freeze-dried kit. 100 µL ^99m^Tc-containing eluate was added. The mixture was incubated for 2 h at 95 °C. To remove loosely bound ^99m^Tc from the ADAPT6 molecules, a 300-fold molar excess of cysteine was added to the mixture followed by additional incubation at 95 °C for 15 min. After the cysteine challenge, the conjugate was purified using disposable NAP-5 size-exclusion column. To determine the radiochemical yield and radiochemical purity, ITLC-SG strips (Agilent Technologies) were used. The ITLC strips were eluted with PBS. The identity of the conjugate was confirmed using gel electrophoresis.

The stability of ^99m^Tc-labeled ADAPT6 was evaluated in the presence of 300-fold excess of cysteine for 1, 2 and 4 h at room temperature and the percentage of released radioactivity from the conjugate was measured using radio-ITLC.

#### *Labeling of DEAVDANS-ADAPT6-GSSC-DOTA with*^111^*In*

Lyophilized DOTA-conjugated (50 µg) ADAPT6 was reconstituted in 120 µL 0.2 M ammonium acetate, pH 5.5. The protein was mixed with 75 µL ^111^InCl_3_ solution (35–70 MBq in 0.05 M HCL) and incubated at 95 °C for 35 min. The labeled construct was challenged with 1000-fold molar excess EDTA at 95 °C for 20 min before purification. The labeled ADAPT6 was isolated using disposable NAP-5 size-exclusion column pre-equilibrated with PBS. In order to determine the radiochemical yield and purity, ITLC-SG developed with 0.2 M citric acid was used and the identity of the construct was analyzed using gel electrophoresis.

The stability of ^111^In-labeled ADAPT6 was evaluated in the presence of 500-fold excess of EDTA for 1 and 2 h at room temperature and the percentage of release radioactivity from the conjugate was measured using radio-ITLC.

### *In Vitro* Evaluation

#### In vitro specificity

Specificity of ^111^In and ^99m^Tc- labeled ADAPT6 was tested using HER2- expressing ovarian carcinoma SKOV3 and breast carcinoma BT474 cell lines by pre-saturation^38^. The cells were seeded into set of six cell culture dishes (5 × 10^5^ cells/dish) two days before the experiment. To saturate the receptors, non-labeled ADAPT6 (2.5 mM) was added to a set of three dishes 15 min before adding the labeled protein.^111^In- or ^99m^Tc-labeled ADAPT6 was added to all six dishes at concentration of 25 nM. After 1 h incubation at 37 °C in a humidified incubator, media was aspirated and cells were harvested after trypsin treatment. Percentage of cell-bound radioactivity for both sets (pre-saturated and unsaturated) was calculated.

#### Affinity determination using LigandTracer

Affinity was measured using previously established method^39^. Briefly 10^6^ SKOV3 cells were seeded on a segment of a cell culture dish. The dish was mounted in a LigandTracer Yellow instrument. Two increasing concentrations of the radiolabeled ADAPTs (0.333, and 1 nM) were used in each affinity assay. The experiment was performed in duplicates. Rates of association to, and dissociation from HER2 expressing SKOV3 cells was recorded and used for affinity determination using the InteractionMap software^39^.

#### Cellular processing

The internalization rate of ^111^In-DEAVDANS-ADAPT6-GSSC-DOTA and ^99m^Tc-DEAVDANS-ADAPT6-GSSC molecules by SKOV3 and BT474 cells during a continuous incubation was evaluated using an acid wash method described earlier^38^. Briefly, cells (5 × 10^5^ cells/dish) were seeded two days before the experiment (three dishes for each time point). Labeled ADAPT6 (25 nM) was added to the cells and incubated at 37 °C in a humidified incubator. At 1, 2, 4, 8 and 24 h, media was aspirated from one set of dishes. To collect the membrane bound tracer, the cells were treated with 0.2 M glycine buffer containing 4 M urea, pH 2.0, and the acid fraction was collected. 1 M NaOH was used to detached the cells and the percentage of radioactivity in the acid fraction and in the cells was calculated.

### *In Vivo* Studies

Animal studies were planned in agreement with Swedish national legislation concerning protection of laboratory animals and were approved by the Ethics Committee for Animal Research in Uppsala. Euthanasia was performed under anesthesia using a combination of Rompun and Ketalar.

BALB/C nu/nu mice were purchased from Taconic M&B. For each time point, a group of four mice was used. Three weeks before the biodistribution study, 10 × 10^6^ SKOV3 cells were subcutaneously implanted in the hind legs of the mice. The average animal and xenografts weights at the time of experiments were 16.5 ± 1.4 g and 0.28 ± 0.09 g respectively. Labeled compounds were injected in tail vein. The injected protein dose was adjusted to 3 μg/mouse by adding non-labeled ADAPT6. The injected activity was 15 kBq for ^111^In and 40 kBq for ^99m^Tc. The total injected volume was adjusted to100 μL. At 1 and 4 h after injection of the radiolabeled ADAPTs, the animals were euthanized by heart puncture, exsanguinated and dissected. Blood and dissected organs were collected, weighed and their radioactivity was measured.

The HER2 specific accumulation in tumors of ^111^In-DEAVDANS-ADAPT6-GSSC-DOTA and ^99m^Tc-DEAVDANS-ADAPT6-GSSC was tested by saturating the HER2 receptors in two groups of mice by injecting of 15 mg trastuzumab per mouse 24 h before injection of labeled ADAPTs. Biodistribution of ^111^In-DEAVDANS-ADAPT6-GSSC-DOTA and ^99m^Tc-DEAVDANS-ADAPT6-GSSC in these mice was measured at 4 h after injection.

To evaluate percentage of ^99m^Tc-DEAVDANS-ADAPT6-GSSC radiometabolites in blood, blood samples were collected 1 h after injection and centrifuged at 30000 rpm for 10 min at 4 °C. Separated blood plasma was loaded onto NAP-5 columns (equilibrated with 1% albumin in PBS) to separate high molecular weight (>5 kDa) fraction from low molecular weight (<5 kDa) fraction. Radioactivity of fractions was measured and percentage of radioactivity associated with low molecular weight catabolites was calculated.

### Imaging

*In vivo* SPECT/CT imaging was performed using nanoScan SC (Mediso Medical Imaging Systems, Hungary) to confirm the biodistribution results. SKOV3 xenografted mice were injected with 3 µg (3 MBq) ^111^In-labeled DEAVDANS-ADAPT6-GSSC-DOTA and 3 µg (7 MBq) ^99m^Tc-labeled DEAVDANS-ADAPT6-GSSC and imaged at 4 h pi. Immediately before imaging, the animals were euthanized by CO_2_ asphyxiation. The CT scans were performed with the following parameters: 480 projections, 50 keV, 670 μA, 2.29 min acquisition time. SPECT helical scans were carried out over 2 h using ^111^In energy window (220.86 keV–269.94 keV; 154.17 keV–188.43 keV) and ^99m^Tc energy window (126.45 keV–154.56 keV), 256 × 256 matrix, 110 projections. CT raw data were reconstructed using Nucline 2.03 Software (Mediso Medical Imaging Systems, Hungary). SPECT studies were reconstructed using Tera-Tomo™ 3D SPECT reconstruction algorithm.
